# Actin cytoskeletal defects in immunodeficiency

**DOI:** 10.1111/imr.12114

**Published:** 2013-10-10

**Authors:** Dale A Moulding, Julien Record, Dessislava Malinova, Adrian J Thrasher

**Affiliations:** 1 Molecular Immunology UnitCenter for Immunodeficiency, Institute of Child Health, University College LondonLondon, UK; 2 Great Ormond Street Hospital for Children, National Health Service TrustLondon, UK

**Keywords:** actin cytoskeleton, immunodeficiency, WASp neutropenia

## Abstract

The importance of the cytoskeleton in mounting a successful immune response is evident from the wide range of defects that occur in actin-related primary immunodeficiencies (PIDs). Studies of these PIDs have revealed a pivotal role for the actin cytoskeleton in almost all stages of immune system function, from hematopoiesis and immune cell development, through to recruitment, migration, intercellular and intracellular signaling, and activation of both innate and adaptive immune responses. The major focus of this review is the immune defects that result from mutations in the Wiskott-Aldrich syndrome gene (*WAS*), which have a broad impact on many different processes and give rise to clinically heterogeneous immunodeficiencies. We also discuss other related genetic defects and the possibility of identifying new genetic causes of cytoskeletal immunodeficiency.

## The actin cytoskeleton

This article is part of a series of reviews covering The Cytoskeleton appearing in Volume 256 of *Immunological Reviews*.

The actin cytoskeleton is composed of a network of actin filaments that are polymerized from actin monomers. Polymerization is initiated by three classes of actin nucleators, the Arp2/3 complex, the formin family, and the more recently identified Spire, cordon-bleu, and leiomodin family (reviewed in 1). Each class of nucleators has a distinct mechanism for initiating actin polymerization. The first actin nucleator to be identified was the Arp2/3 complex 2,3. This complex is unique among all actin nucleators in its ability to form branched actin networks, and its activity is regulated by the eight members of the Wiskott-Aldrich syndrome protein (WASp) family. The next family of actin nucleators identified was the formins, with at least 15 mammalian formins described, and the final family of actin nucleators is the spire, cordon-bleu, and leiomodin family. Nucleation is just one aspect of actin cytoskeletal regulation. Each new actin filament may then be elongated, capped, severed, depolymerized, bundled or crosslinked by actin-binding proteins, and driven by motor proteins such as myosin II (reviewed in 5).

The actin cytoskeleton forms the basis of the cell cortex, onto which the cell membrane is attached and within which microtubules and intermediate filaments form a network that organizes internal cell structure. Restructuring the cytoskeleton allows cells to rapidly change their shape in a timescale of seconds (reviewed in 6). Given that the actin cytoskeleton provides mechanical support for the cell and is the main determinate of cellular rheology 7, the network is surprisingly dynamic, with actin turnover occurring with a half-life as short as 15 s 8. Other components of the actin cortex are even more dynamic; the muscle in the system, myosin II, can turn over in under 10 s 9–10, while some of the proteins that crosslink actin filaments may turn over as rapidly as every second 11. The actin cytoskeleton is a complex system that provides a structure that delivers robust mechanical support yet is sufficiently dynamic to drive cellular morphogenesis. Maintaining this multifaceted functionality requires the regulated interaction of over 100 associated proteins 5.

Cytoskeletal rearrangement generates the driving force for morphogenesis that is essential for cell functions such as mitosis and cytokinesis, cell migration, and intercellular interactions. These processes are central to the development and function of many cell types, tissues, and organs. The critical role of the actin cytoskeleton is evident from the profound impact mutations of actin regulatory proteins have on development, with loss of function of many of these genes resulting in a lethal phenotype. Indeed, if we take actin polymer nucleation as an example, we find that very few of these genes are dispensable in normal development. Loss of Arp2/3 function through knockout of *ARPC3* is embryonic lethal 12. Disruption of the WASp family of Arp2/3 activators is similarly catastrophic: loss of N-WASp, Wave2, and Wash are all embryonic lethal 13,14, and *WAVE1* knockout results in severe neurological defects 16. Loss of the formins Fmn2 or Fhod3 is lethal 17–18, and knockout of other formins, such as Fmn1, result in severe developmental abnormalities 19. The final class of actin nucleators, the WH2-containing proteins, again show severe consequences when their function is lost, with knockout of Spire1 or Spire2 individually showing no phenotype but double knockout resulting in meiosis failure 20.

## Actin cytoskeletal defects as a cause of immunodeficiency

Given the indispensable nature of the actin cytoskeleton, it is perhaps surprising that cytoskeletal defects exist that can cause immunodeficiency without having a far wider impact on development. However, a small number of actin regulatory proteins function solely or primarily in cells of the immune system, and mutation of these genes gives rise to a distinct subset of primary immunodeficiencies (PIDs). Defects in immune function that result from actin cytoskeletal defects encompass nearly every stage of the immune response: proliferation of hematopoietic cells in the bone marrow, migration, and cellular interactions needed to develop into mature effector cells, trans-migration through the endothelium to the sight of infection, dramatic shape change needed to phagocytose invading pathogens, internalization and presentation of antigens, and the intimate cellular interactions needed for direct cell to cell signaling. The first described and most studied actin-related PID is WAS. Through the study of this and other actin-related PIDs, we have made substantial progress in our understanding of the role of the actin cytoskeleton in functioning of the immune system.

## Genetic basis of Wiskott-Aldrich syndrome

WAS (recently reviewed in 21–24) was first described by Alfred Wiskott in 1937 as a syndrome affecting three brothers characterized by abnormally low numbers of small platelets (microthrombocytopenia), bloody diarrhea, eczema, recurrent fever, and ear infections. In 1954, Robert Aldrich described a similar condition over six generations of a single family that affected only men, clearly demonstrating X-linked inheritance. The gene responsible was identified in 1994, located on the X-chromosome 25, and is the founding member of the WASp family of Arp2/3 regulators.

## WASp family proteins

The WASp family regulates actin polymerization through activation of the Arp2/3 complex. There are eight members of this family: WASp; Neural WASp (N-WASp or Wiskott-Aldrich syndrome like, WASL); the three WASp family verprolin-homologous proteins (WAVE/SCAR/WASF 1, 2 and 3); WASp and SCAR homolog (WASH); WASp homolog associated with actin, Golgi membranes, and microtubules (WHAMM); and junction-mediating regulatory protein (JMY). These proteins have no intrinsic catalytic activity and act through a conserved C-terminal domain to activate the ARP2/3 complex. Expression of WASp is restricted to the hematopoietic system 25, WAVE1 and WAVE3 are restricted to neural tissue 26, and the other WASp family proteins are widely expressed 15–29.

## WASp structure and function

**Figure 1:**
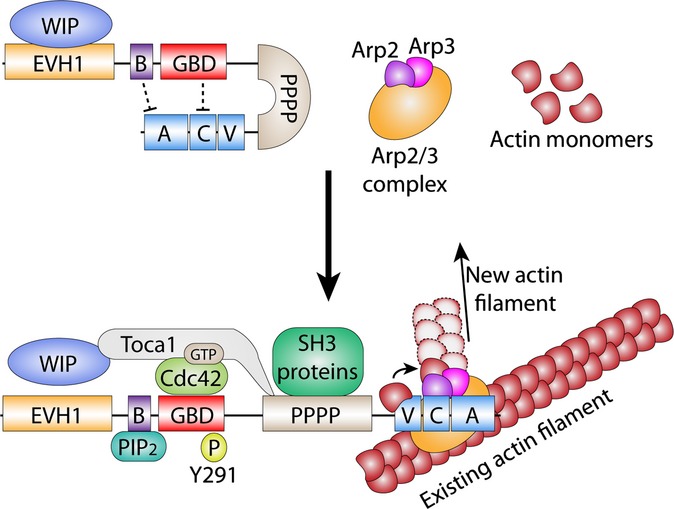
WASp domain structure, interacting proteins, and activation. Cytosolic WASp exists in an auto-inhibited conformation, with the VCA domain tethered to the GBD and basic domains. This inactive state is stabilized by WIP binding to the EVH1 domain. WASp is activated by a variety of signals, including GTP-Cdc42, PIP2, and Y291 phosphorylation by SH3 kinases recruited by the polyproline domain. Toca1 aids WASp activation by displacing WIP, binds GTP-Cdc42, and is required for PIP2 activation of WASp. Activation is restricted to the cell cortex where PIP2 and GTP-Cdc42 are present. Upon activation, the VCA domain is free to bind to and activate Arp2/3. Active Arp2/3 then attaches to an existing actin filament, where Arp2 and Arp3 form the template for a new actin filament branched at a 70° angle from the parent filament.

WASp is a multidomain protein that integrates signals from a variety of intracellular signaling molecules to facilitate the controlled activation of the Arp2/3 complex (*Fig. *1). The five domains of WASp are the N-terminal Ena-VASP homology domain (EVH1), a basic domain, the GTPase binding domain (GBD), the polyproline domain, and finally the C-terminal verprolin homology (also known as Wasp Homology 2) – central – and acidic regions that comprise the VCA domain. WASp shares extensive homology with N-WASp, with 46% identity and 72% homology at the amino acid level and 80% identity in the functional domains. These proteins can substitute for one another in many *in vitro* assays, and biochemical analysis of WASp and N-WASp has often been performed on N-WASp, with WASp function extrapolated from these studies.

Cytosolic WASp is held in an auto-inhibited, inactive conformation through intramolecular tethering of the VCA domain to the central GBD domain. At the cell membrane, WASp is activated by releasing the VCA domain from this inhibited conformation (*Fig. *1). WASp activation has classically been described as relying on Cdc42, phosphatidylinositol 4,5-bisphosphate (PIP2) and tyrosine phosphorylation, with additional input from a range of kinases, adapters and actin-binding proteins(reviewed in 1,22). The combination of these inputs allows for multilevel regulation of WASp, and our understanding of WASp regulation continues to evolve. For example, recent studies have shown how the physical micro-environment can influence WASp activity. WASp dimerization induced by recruitment into large complexes greatly enhances activity, and membrane curvature allows a switch in the activating phosphoinositides from PIP2 to phosphatidylinositol 4-phosphate PI(3)P 30–31.

We are just beginning to understand some of the mechanisms that control WASp inactivation and proteolysis, revealing important roles for WASp stabilization by WIP 32–35 and control of proteolysis by phosphorylation of tyrosine 291. This phosphorylation appears to target WASp for proteosomal and/or calpain-mediated degradation and is important for podosome disassembly and regulation of WASp activity in the IS in T cells 36–39. How Y291 phosphorylation regulates both activation and deactivation of WASp is not fully determined, but one effect appears to be recruitment of the E3 ligase Cbl-b, which contributes to ubiquitination of specific sites in the WH1 domain leading to degradation 37. So, tyrosine phosphorylation may contribute both to activation of WASp and at the same time make it susceptible to proteolytic destruction. This could provide a control mechanism for temporal and spatial limitation of actin polymerization.

## *WAS* mutations

There are over 300 unique disease-causing mutations in *WAS* 21–42 that result in three distinct conditions, classical WAS, X-linked thrombocytopenia (XLT), and X-linked neutropenia (XLN). These 300 mutations are unlikely to be an exhaustive list, as new disease-causing mutations are regularly identified 43. The majority of *WAS* mutations are in the EVH1 domain 40 and result in decreased affinity for the WASp chaperone WIP and reduced WASp expression due to proteolysis 32–46.

Classic WAS tends to result when the mutation leads to near complete loss of WASp or expression of a truncated protein. XLT is a milder form WAS and is often a result of *WAS* mutations that allow residual although substantially reduced WASp expression. Some mutations, however, may allow expression of normal amounts of WASp that is functionally defective, for example through disturbance of Arp2/3 complex binding (A. J. Worth, unpublished data, Austen Worth, personal communication). X-linked neutropenia is quite different from both WAS and XLT and is the result of a mutation within the GBD that disrupts the auto-inhibited conformation of WASp, generating a constitutively active protein. Four distinct mutations have been described that cause XLN (L270P, S272P, I294T, and I276S), although others in the same region would be expected to produce a similar functional effect 22–49.

The clinical presentation of the mildest form of disease, XLT, may be restricted to microthrombocytopenia or may present as a slightly more severe form with mild transient eczema and occasional mild infections. Classical WAS presents as microthrombocytopenia with persistent eczema and recurrent infections, which vary in severity and frequency in different patients and may be frequently complicated by autoimmunity and in some cases lymphoid malignancy. XLN presents as neutropenia with recurrent bacterial infections and is discussed in more detail later.

## Immune cell defects in WAS

The complex nature of WAS stems from the multiple functional defects that occur in nearly all immune cells and almost every step of the immune response, although to varying extents. Here we discuss these defects by cell type and immune compartment to present an overview of our current understanding of how these defects combine to produce the multi-lineage disease phenotype, with immunodeficiency, autoimmunity and susceptibility to malignancy.

### Hematopoiesis and homeostasis

WASp is expressed from the very earliest stages of the hematopoietic system in the developing embryo 50. Apparent non-random X-chromosome inactivation in WAS carrier female patients points to a vital role in hematopoietic cell homeostasis and survival 51. During development, the hematopoietic system relocates from the fetal liver to the bone marrow. Fetal liver hematopoietic cells in carrier female mice have random X-chromosome inactivation, but bone marrow cells have non-random inactivation of the defective WAS X-chromosome. Therefore, WASp positive cells have a selective homing advantage 52. However, WAS patients and mouse models show normal bone marrow cell numbers and proliferation, suggesting partial redundancy between WASp and other WASp family members in the developing hematopoietic system. While WASp deficiency has only a small impact on hematopoiesis, the numbers of certain subsets of peripheral immune cells, especially T cells, show an accelerated decline through childhood 53–54. In experimental systems (and recently in clinical gene therapy studies), a clear selective advantage for survival of mature hematopoietic cells encompassing CD4^+^ and CD8^+^ T cells, regulator T cells (Tregs), natural killer (NK) cells, circulating B cells, splenic B cells, splenic macrophages, NK cells, and platelets has been demonstrated 55–56. This may contribute to the observed high number of different somatic revertants found in WAS patients, although other undetermined molecular mechanisms are also likely to be important. Somatic reversions are generally found in the mature T-cell and NK-cell compartments 23, and although some functional recovery would be expected, it has been difficult to identify consistent clinical benefit, perhaps highlighting the multi-lineage nature of the disease 57. It may also depend on the diversity of functional T cells generated by reversion and the stage of differentiation and development at which the cells accumulate.

### Platelets

Microthrombocytopenia is consistently present in WAS and XLT. The resulting tendency for bleeding is a significant cause of morbidity and mortality 58. WASp expression in platelets is extremely low, even when other cell types have residual WASp expression, possibly because once produced, anucleate platelets have a limited capacity to synthesize protein 59. Where mutant WASp expression has been demonstrable in platelets, there was correlation with a milder level of microthrombocytopenia 60. Despite the invariable microthrombocytopenia in WAS and XLT, our understanding of the role of WASp in platelet biogenesis and function is rather limited. Platelet biogenesis begins in the bone marrow, where megakaryocytes extend pro-platelet elongations that are then released into the vasculature where they continue to mature and finally release platelets 61. WASp null megakaryocytes have abnormal actin cytoskeletal architecture, but despite a premature release of pro-platelets *in vivo* 62, *in vitro* studies show WAS megakaryocytes generate pro-platelets and platelets of normal size and numbers 63. Thrombocytopenia in WAS actually appears primarily to be due to increased platelet clearance, even in the absence of concurrent autoimmunity. WASp null platelets are cleared more rapidly than normal platelets when transferred to wildtype mice, due to increased phagocytosis by splenic, bone marrow, and liver macrophages, and compounded by autoimmune anti-platelet antibodies 64,65. Consistent with the idea that increased clearance is a major factor in microthrombocytopenia, splenectomy has been shown to give a consistent and marked improvement in both platelet number and size, although thrombocytopenic relapse may occur in the presence of anti-platelet antibodies 67–70. WAS platelets are small and misshapen in non-splenectomized patients but show a remarkably normal size, shape, and function in splenectomized patients (reviewed in 21).

### Innate immunity: myeloid cells

Myeloid phagocytic cells of the innate immune system form the frontline of defense against invading pathogens. These cells detect and migrate toward pathogens, release signaling molecules, produce toxic metabolites, phagocytose, and destroy their targets. Studies of WAS patient-derived cells and animal models demonstrate how an inability to reorganize the actin cytoskeleton impacts on all of these processes.

WAS phagocytic cells have a poor chemotactic response toward inflammatory chemoattractants such as bacterially derived fMLP, and the chemokines c5a, MCP-1, and CSF-1 53–73. These migration defects are most apparent under conditions of shear flow or when studied *in vivo* in WAS knockout mice or WAS knockdown zebrafish embryos 73,74. The failure to polarize the cytoskeleton and direct integrin clustering has knock on effects, such as reduced levels of neutrophil oxidative burst and degranulation 75.

Mechanistically, the lack of WASp prevents cytoskeletal rearrangements, including podosome formation 75–78, that are required to guide adapter proteins such as vinculin and talin to physically induce integrin clustering necessary for adhesion to surrounding tissues 79–80. WASp-deficient cells are unable to maintain directional protrusions during migration 81 and lose cellular polarity between the leading pseudopod and trailing uropod due to failure to direct integrin CD11b to the uropod where it stabilizes microtubules 82. Despite these migration defects, some cells do successfully migrate to the target site but show defective phagocytosis of pathogens 83–84 and apoptotic cells 85–86. These phagocytic defects result from compromised actin-driven phagocytic cup formation 79, which normally relies on phospholipase D2 and Grb2 binding to WASp 87.

### Innate immunity: lymphoid NK and iNKT cells

NK cells are lymphoid innate cells that migrate toward sites of infection and inflammation, where they detect virally infected cells, parasites, and malignant cells, and kill them via the release of cytotoxic proteins 88. The activation of NK cells is severely compromised in WAS, as it relies on the formation of an immune synapse, which is dependent on WASp mediated actin polymerization 89. Surprisingly, NK cell function and immune synapse formation can be restored in the presence of interleukin-2 (IL-2) 90 through activation of WAVE-2, even in the absence of WASp 91.

Invariant NKT (iNKT) cells blur the boundary between innate and adaptive immunity, expressing receptors typical of both NK cells and T cells. They recognize lipid antigens presented via the non-classical major histocompatibility complex (MHC) class I molecule CD1d and possess an invariant T-cell receptor α chain 92. iNKT cells act early in the immune response, release a wide variety of cytokines, including IL-4 and interferon-γ (IFNγ), and appear to have important functions in antitumor activity and autoimmunity 92–93. The low numbers of iNKT cells together with defective function of NK cells 94–95, may well provide an important contribution to disease phenotype.

### Antigen presentation: dendritic cells

The interaction between the professional antigen-presenting cells of the innate immune system and lymphocytes of the adaptive immune system represents a major focus of research in WAS. These studies highlight the variety of defects that a loss of actin cytoskeletal rearrangement can cause.

Migration of dendritic cells (DCs) toward sites of inflammation is impaired *in vivo* due to failure to polarize. Lack of podosome formation and poor integrin assembly reduce the adhesion to intercellular adhesion molecule-1 (ICAM-1) needed for migration over the endothelium 80–100. DC uptake of soluble antigen is normal in WAS, but phagocytosis and presentation of particulate antigens is impaired 101. The migratory defects in WAS DCs may also contribute to failure of DCs to correctly localize in secondary lymphoid tissues, with dyslocalization seen in both lymph nodes and spleen 98–99. Poor DC migration after antigen uptake may lead to maturation of DCs before they reach lymph nodes, with ectopic cytokine and chemokine release likely to recruit other immune cells that may contribute to inflammatory processes such as eczema.

The reduced numbers of DCs that do arrive at the lymph nodes have a depleted ability to form an immune synapse and present antigen. This defect is very well characterized on the lymphocyte side (see below), but is also due to specific defects in the DC. Activation of normal (expressing wildtype WASp) T cells and NK cells by WAS DCs is impaired both *in vitro* and *in vivo* 100,102. This is a result of reduced IL-12 release from the DC and also from poor immune synapse formation 104. In these experiments, although T cells had normal WASp activity, these T cells failed to efficiently polarize T-cell receptors (TCRs), LFA-1, talin, and F-actin, resulting in diminished TCR signaling and demonstrating that DC-specific defects significantly contribute to WAS. For example, a DC-specific defect of type I IFN production could explain compromised antiviral immunity 105.

### Adaptive immunity: T cells

Thymic T-cell development is surprisingly intact in WAS, although detectable abnormalities do exist 106–107. Mature WAS T cells exhibit morphological changes, with fewer microvilli, but the significance and impact of these changes are not clear 108–109. WAS T cells have a marked migration deficiency, with poor adhesion and failure to respond to the chemokines CCL19 and CCL21^73^. A major defect in WAS T cells is their failure to proliferate in response to immune activation following TCR ligation and in the context of an immune synapse (IS) 53–111. Abnormal IS formation results from actin polymerization defects, failure to mobilize lipid rafts, and poor assembly of the signaling partners that normally rely on WASp for localization to the TCR 112–113. The IS is a highly dynamic structure that cycles through stable symmetrical and unstable asymmetric forms, with WASp required for stable IS reformation after protein kinase Cθ (PKCθ)-triggered IS disruption 114. Therefore, while the initial formation of the IS may be reasonably normal in WAS, it is inherently unstable and cannot maintain symmetry. In the absence of WASp, the IS can form normally if there is strong costimulation and antigen concentration is high 115. This observation suggests WASp function in IS formation may be redundant given sufficient costimulation. However, signaling downstream of TCR ligation is consistently reduced due to failure to translocate the transcription factor nuclear factor of activated T cells (NFAT) to the nucleus. This translocation requires the EVH1 domain of WASp and is independent of WASp Arp2/3 activation 116–117. These signaling failures mean WAS CD4^+^ T cells fail to secrete normal amounts of the T-helper 1 (Th1) cytokines IFN-γ and tumor necrosis factor-α (TNF-α), but Th2 cytokine (IL-4, IL-5, and IL-10) production is near normal 116.

An unanticipated role for WASp in the nucleus has been reported that may provide an explanation for the Th1-Th2 imbalance in WAS 118. In this study, WASp was shown to localize specifically to the Th1 master gene *TBX21* and is necessary for *TBX21* transcription through an epigenetic mechanism involving histone methyltransferase recruitment. Interestingly, a WASp mutant found in the mildest form of XLT retained *TBX21* localization and permitted normal *TBX21* transcription.

### Adaptive immunity: B cells

Despite clear humoral immune defects, low immunoglobulin M (IgM) levels, elevated IgA and IgE, and increased incidence of B-cell malignancy 119, B-cell function in WAS has received much less attention. Initial reports showed little impact of WASp deficiency in B cells 84,106, with the exception of a conflicting report of failure of Epstein–Barr virus (EBV) immortalized WAS B cells to proliferate 121. B-cell defects were limited to decreased antibody responses to polysaccharides 54–119 and fewer microvilli 84. However, more detailed analysis of B cells in WAS revealed that these cells did have marked abnormalities, including defective polarization, spreading, aggregation, and migration toward CXCL13 *in vitro*, few microvilli, and delayed humoral immune response in mice 122–123. This was supported by the finding of poor adhesion, abnormal actin cytoskeletal architecture, defective IS formation, and augmented apoptosis in human pre-B cells 55–124. Conditional depletion of WASp in B cells also results in hyperproliferation 125–126. Splenic architecture is abnormal in WAS mice and humans, but marginal zone B-cell numbers are characteristically low, perhaps due to failure to migrate toward sphingosine-1-phosphate and CXCL13 36–128. B-cell defects are most pronounced in mature B cells, where WASp expression is normally highest 55, with developing B cells able to partially compensate possibly through N-WASp 129. Autoantibody production is a common feature in WAS 119,130, although the mechanisms behind this are poorly understood.

### Autoimmunity and malignancy

Autoimmunity is common in classical WAS, eventually affecting the majority of patients if untreated by hematopoietic stem cell transplantation (HSCT) or more recently gene therapy 132. Management of autoimmune manifestations is becoming increasingly important as the treatment of immunodeficiency and thrombocytopenia improves 119–134. Antibody-mediated cytopenias are the most common autoimmune presentation, followed by vasculitis, arthritis, colitis, and nephritis 22. Autoimmunity can occur or persist even after successful HSCT and may correlate with the level of chimerism achieved 133–134. Recent studies have begun to unravel the causes of autoimmunity in WAS, but our knowledge in this area is limited to a few key studies and will likely be an area of active research for some time. Autoimmunity is thought to result from a breakdown of tolerance to self-antigens, giving rise to autoreactive T and B cells. Tolerance is maintained by Tregs 135 and also the recently described regulatory B cells (Bregs) 136–137. There was very little mechanistic understanding of autoimmunity in WAS until four reports published in 2007 demonstrated a variety of functional defects in Tregs, even though defects of apoptotic cell clearance had been described 85–141. WAS Tregs fail to proliferate after TCR activation, show lower levels of activation markers such as CD25, produce less immunosuppressive IL-10, lack tissue homing markers (potentially explaining the very low numbers of Tregs at sites of inflammation and lymph nodes), and have reduced suppressive activity both *in vitro* and *in vivo*. The loss of Treg function is partly attributable to the low levels of CD25 (part of the IL-2 receptor) and addition of exogenous IL-2 can partially restore Treg function *in vitro*. This is likely compounded by the lack of IL-2 production from WAS effector T cells, although *in vivo* the presence of wildtype effector T cells with normal IL-2 secretion is unable to prevent autoimmunity 141. The loss of Treg activity also impacts on B-cell tolerance, as WAS Tregs are inefficient in preventing B-cell proliferation, and show reduced killing of B cells, poor polarization, and little secretion of the apoptosis inducing protein granzyme B 142. Another contributing factor is a reduction in restimulation-induced cell death (RICD) in WAS. RICD is a process that acts to remove T cells responsive to persistently expressed antigens such as autoantigens. In WAS, RICD-induced apoptosis is reduced in CD4^+^ T cells, possibly because of low production of vesicular FasL 143.

WASp deficiency also plays a B-cell-intrinsic role in the production of autoantibodies. Removal of self-reactive B cells is largely dependent on the strength of the BCR signal 144–145. Mouse WAS B cells are hyper-responsive to B-cell receptor and Toll-like receptor signaling, leading to a loss of immune tolerance 125. The kinetics of B-cell receptor internalization after engagement are much slower in WAS B cells, possibly due to defective cytoskeletal rearrangement 125. However, while WAS mice show elevated levels of autoantibodies, they show little sign of autoimmunity, possibly due to the wider WAS immunodeficiency and particularly the lack of T-cell help 24–125. However, when WASp deficiency is restricted to B cells by conditional knockout of WAS in the B-cell lineage, mice develop severe autoimmunity, spontaneous germinal center formation, hyper-proliferation of germinal center B cells and plasma cells *in vivo*, and excessive differentiation of class switched plasmablasts *in vitro* 125–126. The role of IL-10 producing Breg cells has not been properly explored but may well also be important for control of autoimmune processes 136–137.

WAS patients have an increased risk of malignancy, particularly B-cell lymphoma. Inefficient killing of B-cell lymphoma targets by WAS cytotoxic T lymphocytes, owing to poor polarization and delivery of cytotoxic granules to the T-cell/target cell interface and also lower cytokine (IL-2, IFN-γ, and TNF-α) production may contribute, together with functional deficiency of NK and NKT cells 146. Malignancy risk in WAS is another area that needs further investigation and will pose a greater problem as long-term disease management improves. Overall, the mechanisms behind susceptibility to malignancy are incompletely understood, in terms of intrinsic cellular contribution as well as systemic immunodeficiency.

## Other actin-related immunodeficiencies

*WAS* is the first in a growing list of genes whose disruption result in immunodeficiencies that are due to cytoskeletal abnormalities. These genes show a range of activities and interactions with the cytoskeleton, including a mutation in actin itself, actin bundling, actin severing, inhibition, and promotion of actin polymerization, signal transduction, and transcription (*Table *1).

**Table 1:** Actin cytoskeletal immunodeficiency: phenotypes and candidate genes

Protein (*gene*)	*Expression profile* and function	Disease or mouse model	Phenotype
WASp – Wiskott-Aldrich Syndrome Protein *(WAS)*	*Hematopoietic*. Arp2/3 activator. Branched F-actin network assembly. Transcription factor activation. Epigenetic chromatin remodeling	Wiskott-Aldrich Syndrome (WAS) X-Linked Thrombocytopenia (XLT) X-Linked Neutropenia (XLN)	Severe combined immunodeficiency. Autoimmunity, malignancy. All immune lineages affected. Multiple cytoskeletal cellular defects. Thrombocytopenia, mild immunodeficiency. Neutropenia, excessive F-actin production. Cell division defects
WIP – Wiskott-Aldrich Syndrome Protein Interacting Protein (*WIPF1)*	*Hematopoietic*. WASp chaperone. Prevents WASp proteolysis, aids WASp recruitment, and regulates activity.	Novel human immunodeficiency	Identical to WAS
Dock8 – Dedicator of cytokinesis 8 *(DOCK8)*	*All tissues, strong hematopoietic expression*. Atypical guanine exchange factor. Signaling from surface receptors to cytoskeletal regulators, signal transduction	Dock8 deficiency. Autosomal recessive hyper-IgE syndrome (AR-HIES)	Severe combined immunodeficiency. Autoimmunity, malignancy. All immune cell lineages affected? Multiple cytoskeletal cellular defects. Signal transduction impaired in B-cell proliferation (and other cell types?)
Rac2 Ras-Related C3 Botulinum Toxin Substrate 2 *(RAC2)*	*Hematopoietic*. Rho GTPase, signal transduction, cytoskeletal remodeling	Neutrophil immunodeficiency syndrome	Neutrophil chemotaxis, actin polymerization, oxidative burst. Mouse: Low thymic output, HSC apoptosis, Poor T-cell activation and proliferation, B-cell activation, migration
RhoH ras homolog family member H *(RHOH)*	*Hematopoietic*. Atypical Rho GTPase, signal transduction, cytoskeletal remodeling	Epidermodysplasia verruciformis	Increased memory T cells, restricted TCR usage. Persistent EV-HPV infection. Mouse: Poor HSC proliferation, dysregulated F-actin production. T-cell deficiency, T-cell development, TCR signaling, migration
Coronin 1A coronin, actin-binding protein, 1A *(CORO1A)*	*Hematopoietic*. Arp2/3 inhibitor	Severe combined immunodeficiency with CORO1A mutation	Naive T-cell deficiency, defective egress from the thymus, reduced TCR repertoire, lack of iNKT cells. Mouse: increased F-actin in T cells, increased T-cell apoptosis, TCR signaling defect
β-Actin *(ACTB)*	*All tissues*. Main Actin protein	A unique neutrophil dysfunction	Poor neutrophil chemotaxis, oxidative burst and actin remodeling. Thrombocytopenia
Leukocyte specific protein 1 (*LSP1*)	*Hematopoietic*. Actin bundling	Neutrophil actin dysfunction syndrome (NAD47/89)	LSP1 overexpression impedes neutrophil and macrophage migration and phagocytosis
L-Plastin *(LCP1)*	*Hematopoietic*. Actin bundling	Mouse model, cell lines	Multiple hematopoietic lineages affected. Oxidative burst, signaling, motility, adhesion defects reported
RhoG ras homolog family member G *(RHOG)*	*Hematopoietic*. Rho GTPase, signal transduction, cytoskeletal remodeling	Mouse model	Increased IgG1 and IgG2B, small increase B and T-cell proliferation after activation. Macrophage phagocytosis defective, oxidative burst defect, TCR capping and internalization defect
AIP1/WDR1 – Actin-Interacting Protein 1/WD repeat domain 1 *(WDR1)*	*All tissues*. Enhances actin depolymerization in cooperation with cofilin	Mouse model, cell lines	Autoinflammatory disease. Excessive neutrophil numbers at inflammation sites. Excess F-actin, defective migration
MKL1/MAL/MRTF-A – Megakaryoblastic Leukemia 1 *(MKL1)*	*All tissues*. Senses the G-actin pool, transcriptional co-activator of SRF, control many actin-related genes	Mouse models	Megakaryocyte development perturbed. Thrombocytopenia

### X-linked neutropenia

XLN is characterized by severe neutropenia and monocytopenia, a marked reduction in NK cells, skewed CD4^+^/CD8^+^ T-cell ratios, and recurrent bacterial infections 49. Surprisingly, the gene identified as causing XLN is *WAS*, with four mutations described, L270P, S272P, I276S, and I294T, clustered together in the GBD of WASp 22–49. These mutations disrupt the auto-inhibited conformation of WASp, generating a constitutively active protein. Consequent activation of the Arp2/3 complex triggers uncontrolled actin polymerization and an increase in cellular F-actin content.

At the cellular level, defective cytoskeletal dynamics in XLN result in abnormal phagocytosis, migration, and podosome dynamics 47. Although oxidative burst in neutrophils is normal in response to PMA, receptor-mediated oxidative burst in response to *E. coli* or fMLP is reduced, suggestive of an inability to effectively assemble signaling complexes at the cell membrane 47. In addition to cytopenias and myeloid defects in XLN, T-cell proliferation is reduced in response to CD3 stimulation, and spontaneous apoptosis of bone marrow-derived progenitor cells and Fas-mediated apoptosis of lymphocytes are increased 47. B-cell morphology is abnormal, B-cell rolling on L-selectin is impaired 147, and B and T-cell spreading is reduced 148.

*In vitro* studies using forced overexpression of WASpI294T showed that the reduced proliferation and cytopenias in XLN are caused by ectopic F-actin impeding mitosis and cytokinesis, leading to apoptosis and genomic instability. These cell division defects are a result of altered cell mechanics. Constitutively active WASp is active throughout the cytoplasm, so branched F-actin networks, normally restricted to the cortex, form throughout the cell. The resulting network of cytoplasmic F-actin increases cellular viscosity and elasticity 7–149. These changes in cell mechanics directly impede cell division by resisting the movement of mitotic chromosomes and by slowing the closure of the actomyosin ring during cytokinesis 149–150. The cell responds to these mitotic abnormalities through the activity of a cell cycle regulating kinase, Aurora B, and also compensates for increased viscosity by strengthening the mitotic spindle. Despite these compensatory mechanisms, division defects frequently occur in XLN cells. In addition to the genomic instability observed *in vitro,* evolution to myelodysplasia or AML with acquisition of *CSF3R* mutations and monosomy 7 has been reported in XLN patients 47–151, and aneuploidy is also evident in a mouse model of disease 148.

### WIP deficiency

Female carriers of *WAS* gene mutations can develop clinical manifestations of disease if X-inactivation is sufficiently skewed. A female patient with the classical symptoms of WAS, including eczema, thrombocytopenia, recurrent infections, defective T-cell proliferation and chemotaxis, and impaired NK cell function was found to have normal WAS gene sequence and mRNA levels but a complete absence of WASp expression 152. Because WIP stabilizes WASp 32,33, WIP expression was assessed. WIP protein was reduced by approximately 50% in both parents, and completely absent in the patient. *WIPF1* gene sequencing revealed a homozygous nonsense mutation in the patient, resulting in a truncated 435 amino acid WIP protein lacking the WASp binding domain (aa 451–485) 153. This novel route to WASp deficiency should be considered in patients that present with WAS phenotype but have a normal *WAS* gene.

### DOCK8

Dedicator of cytokinesis 8 (DOCK8) was isolated in a yeast two-hybrid screen for proteins interacting with Cdc42, and DOCK8 also binds to Rac1, RhoJ, and RhoQ 154. DOCK8 mRNA is present throughout most tissues, with strong protein expression in hematopoietic cells and peripheral blood mononuclear cells (PBMCs). DOCK8 localizes to cell edges during lamellipodia formation 154, and is part of the Dock180-related family of atypical guanine exchange factors (GEFs). GEFs in general activate Rho GTPases by facilitating the switch from a GDP-bound to GTP-bound state. The Rho GTPases, in turn, integrate extracellular signals and transduce these to effector molecules (i.e. GTP-Cdc42 activates WASp) to produce an appropriate cytoskeletal response 155.

In 2009, DOCK8 mutations were independently described as the cause of severe immune deficiency in both humans and mice 156,157. The syndrome was originally described as an autosomal recessive form of hyper IgE syndrome (AR-HIES), due to several shared features, such as elevated IgE, respiratory infections, and eosinophilia. However, using high-resolution comparative genomic hybridization, Zhang *et al*. 157 discovered homozygous or compound heterozygous deletions and point mutations in *DOCK8* in 11 patients who had previously been diagnosed with AR-HIES or unknown combined immunodeficiencies. It has since been shown that *DOCK8* mutations account for the majority of cases of AR-HIES with over 60 patients now reported in literature 157–167.

Clinical characteristics of DOCK8 deficiency include severe food or environmental allergies, otitis media, pneumonia, or bronchitis, eczema, eosinophilia, IgE dysregulation, and severe cutaneous viral infections, with the most common culprits being human papilloma virus (HPV), molluscum contagiosum virus, herpes simplex virus, and varicella-zoster virus 157,158. Systemic viral infections are rarely detected, suggesting defects in antiviral immunity locally within the skin 158–168. Susceptibility to viral infections is seen in other PIDs such as WAS and ‘leaky’ SCID 169,170. As in WAS, the increased susceptibility to viral infections could be the result of a combination of factors including defective skin barrier, reduced numbers of T cells, impaired T-cell proliferation and antiviral cytokine production, and abnormal migration into infected tissues 172. Pneumonias in DOCK8 patients are caused by a wide spectrum of Gram-positive and Gram-negative bacteria and fungi. Gastrointestinal tract infections, including *Giardia* and *Salmonella*, have also been reported 157–158.

DOCK8 is a key regulator of actin cytoskeletal dynamics, and as such DOCK8 deficiency elicits a broad range of immune cell defects. Despite the distinct clinical presentation of DOCK8 compared to WAS, many of the cellular defects are shared between these two syndromes. T-cell defects common to DOCK8 deficiency and WAS include decreased proliferation *in vitro* 157–158 and fewer memory T cells *in vivo* 173–174. Both syndromes show decreased antiviral cytokine (IFN-γ and TNF-α) production by CD8^+^ T cells, although in DOCK8 deficiency granule release appears normal 157. The Th1-Th2 imbalance in WAS might also be a feature of DOCK8 deficiency, as some patients have elevated levels of IL-6 and IL-10 162. DOCK8 deficiency also shows T-cell defects not described in WAS. Low numbers of Th17 cells in DOCK8 deficiency 175–176 may contribute to poor antifungal immunity. There is also evidence of impaired T-cell production or efflux from the thymus 162. It is still not clear to what extent defective TCR signaling, cytokine production, and homeostatic proliferation of peripheral cells contribute to the overall T-cell defects. The defective functioning of CD8^+^ T cells, which normally assist in tumor surveillance, may contribute to the higher rate of malignancies in DOCK8 patients. Around 20% of patients develop at least one type of cancer, and the most common malignancies are squamous cell carcinoma and lymphoma 168.

In the B-cell compartment, marginal zone B cells are lacking in both WAS and DOCK8 deficiency, and B cells in both syndromes have poor IS formation due to a failure to concentrate LFA-1 and ICAM-1 in the pSMAC (peripheral supramolecular activation cluster) 55,156. Competitive hematopoietic chimeras have shown that DOCK8 mutant B cells contributed normally to circulating B cells but poorly to the germinal center (GC) subset, in particular during response maturation, suggesting that the mutation causes an intrinsic B-cell defect affecting their ability to differentiate into or sustain a GC fate. DOCK8 mutant B cells also undergo normal immunoglobulin gene hypermutation; however, diminished survival and selection resulted in severely reduced numbers of high-affinity IgG^+^ B cells 156. The high frequency of recurrent infections in DOCK8 patients implies defects in humoral immunity. However, serum IgA levels, important for mucosal immunity, are reduced only in a minority of patients while most show normal or increased titers 157–158, similar to WAS. It is not clear how DOCK8 patients exhibit hypo-IgM and hyper-IgE. However, DOCK8 was recently shown to transduce signals between TLR9 and STAT3 via MyD88, a novel signaling pathway for TLR9-driven B-cell proliferation and immunoglobulin production 177.

There are relatively few studies of other cell type-specific defects in DOCK8 deficiency. DOCK8 DCs show similar defects to those found in WAS. That is, defective cytoskeletal reorganization with decreased migration to lymph nodes, slow velocity through dermal tissues and poor T-cell priming, although antigen uptake and presentation is normal 178. NK cell numbers are normal in DOCK8 deficiency, but unlike WAS, DOCK8 NK cells show normal F-actin levels. However, similar to WAS, DOCK8 NK cells are unable to polarize F-actin toward the lytic synapse, with impaired clustering of CD18, and lack of polarization of perforin and the microtubule organizing center 179. In WAS, cytolytic activity of NK cells can be restored by IL-2 as the cytokine can bypass WASp to use the homologous effector WAVE2 91. This is not the case for DOCK8 NK cells; IL-2 stimulation had no effect on NK cell functions in the absence of this upstream activator 179.

The disease pathology of DOCK8 deficiency is likely to be the result of a combination of cellular defects including the inability of B cells to generate high-affinity antibodies and sustain GCs, the absence of T-cell memory, defects in DC migration, capacity to prime T cells, and reduced NK cell cytotoxicity. In mice, DOCK8 mutations replicate many of the cellular defects described in human disease, but mice show no obvious clinical phenotype, another similarity to WAS 84–106. Some of the symptoms described, such as rash, elevated IgE, and susceptibility to autoimmunity, are very similar to those described in WAS 22. As an effector of the Rho GTPase Cdc42, WASp presumably acts downstream of DOCK8, and the similar clinical manifestations may reflect the overlapping signaling pathways. While WASp function may also be diminished in DOCK8 patients, this cannot explain the complete phenotype of DOCK8 deficiency, and other downstream DOCK8 effectors such as N-WASp may also be involved. Interestingly, mice generated to delete *WAS* and *N-WAS* in lymphocytes show an exaggerated cellular phenotype 107–129.

### Rac2, RhoH, and the Rho GTPase signaling pathway

The 20 mammalian Rho GTPases constitute a highly conserved family of signaling proteins that activate downstream targets including actin nucleators, protein kinases, and phospholipases, to regulate cytoskeletal dynamics, transcription, and proliferation 180–181. In addition to immunodeficiency due to defects in upstream regulators (DOCK8), downstream effectors (WASp) of Rho GTPase signaling, immunodeficiency can also result from mutations in the Rho GTPases themselves.

The Rho GTPase Rac2 is restricted to hematopoietic cells and is abundantly expressed in neutrophils, constituting 96% of the total Rac in these cells 182. *RAC2* KO mice revealed an essential role for Rac2 in neutrophil chemotaxis, L-selectin capture and rolling, F-actin generation, chemoattractant-driven activation of p38 and p42/44 MAPKs, and oxidative burst 183. In humans, *RAC2* mutation is the cause of neutrophil immunodeficiency syndrome 182–184. This remains an extremely rare disorder, with a third case described in 2011 185. All three cases share an identical D57N mutation, within the DX_2_G motif conserved in all GTPases, resulting in a dominant negative-acting protein. Analysis of patient samples showed poor neutrophil chemotaxis, lack of actin polarization, diminished granule secretion, and no oxidative burst in response to fMLP, although oxidative burst is present following direct activation of PKC 182,184. The rarity of *RAC2* mutations has limited its study in a human setting, and only one further cellular defect has been described in human Rac2 disease: impaired thymic T-cell production, possibly related to defective T-cell integrin function 185.

Studies using mouse models of Rac2 deficiency, with dominant negative D57N Rac2 or *RAC2* KO, not only recapitulate the neutrophil defects 186,187 but have also revealed a far wider impact, encompassing almost all aspects of immune function 181. HSCs show increased apoptosis and loss of retention in the bone marrow 186,189. T lymphocytes show reduced activation following TCR activation, defective cytoskeletal reorganization, and lack of Th1 signaling and IFN-γ production 191–192. B-cell development, cytoskeletal reorganization, activation through BCR signaling, and migration to chemokines are all defective 193,194. Macrophages display phagocytic and oxidative burst deficiency similar to neutrophils 196, and the CD8^+^ subpopulation of DCs also lacks oxidative burst capacity 197, while eosinophils have impaired migration and actin polymerization 198.

RhoH is an atypical Rho Family GTPase that lacks GTPase activity and acts by inhibiting other GTPases. RhoH expression is restricted to hematopoietic cells, and knockout mice develop normally but display a range of immune cell dysfunctions. HSC proliferation and migration are perturbed due to excessive Rac1 activity, resulting in enhanced F-actin production, as the normal Rac1 inhibitory activity of RhoH is absent 199–200. T-cell development, TCR signaling and migration are all disturbed, resulting in T-cell deficiency 201,202. Recently a loss of function mutation in *RhoH* was identified as a novel cause of a rare genetic disorder, epidermodysplasia verruciformis, which normally results from mutation of the eponymous gene 204. The two patients described had recurrent infectious diseases, most notably persistent EV-HPV infection. T cells were skewed toward effector memory cells with restricted TCR usage and poor TCR signaling. T-cell homing appears to be deficient due to lack of β_7_ integrin expression, which may explain the persistent EV-HPV infections.

### Coronin 1A

Coronins are actin regulatory proteins conserved in all eukaryotes. In mammals, seven members are expressed and can be classified into three types: I, II, and III. Type I coronins include Coronin 1A, B, and C. Although Coronin B and C are ubiquitous, Coronin 1A is restricted to hematopoietic cells. Type I Coronins regulate actin filament branching by restricting nucleation of branched actin filaments at the leading edge, either by inhibiting the attachment of the Arp2/3 complex or directly facilitating debranching 205. Studies in mice revealed a role for Coronin 1A in actin dynamics in T cells, T-cell homeostasis and trafficking, with a calcium signaling defect downstream of TCR and B-cell receptor activation, resulting in reduced proliferation and IL-2 production 206–209. In humans, Coronin 1A mutations have been reported to cause severe combined immunodeficiency, with severe peripheral T-cell deficiency, especially in naive T cells, near complete absence of iNKT cells, and susceptibility to EBV-induced lymphoproliferation 210,211. Interestingly, in a mouse model of systemic lupus erythematosus (SLE), loss of Coronin 1A activity in T cells completely suppressed the autoimmunity normally present in SLE 209.

### β-Actin

As the main component of the actin cytoskeleton, it is surprising that a non-lethal mutation in the β-Actin gene (*ACTB*) has been reported that results in immunodeficiency 213. The patient presented with recurrent infections, thrombocytopenia, intellectual impairment, and short stature. A heterozygous mutation in *ACTB* resulting in an E346K substitution was identified, in a region of the protein that is reported to bind a number of cytoskeletal regulators. The major immune defect was poor neutrophil chemotaxis and oxidative burst and failure to polarize the cytoskeleton in response to fMLP. No other immune dysfunction was reported. Further studies of this mutation have not yet been performed.

### Leukocyte-specific protein 1

Leukocyte-specific protein 1 is an actin-binding phosphoprotein mainly expressed in neutrophils, macrophages, B and T lymphocytes, and endothelial cells 214. It binds actin through caldesmon and villin homologous actin-binding domains, reorganizes actin filaments into bundles, and localizes to F-actin in lamellipodia, filopodia, and membrane ruffles 215–218. A mouse knockout model for *LSP1* demonstrated increased migration of neutrophils and macrophages toward inflammatory sites, and enhanced chemotaxis toward fMLP and chemokines, indicative of a negative regulatory role of LSP1 in chemotaxis 219. LSP1 was identified as a 47 kDa protein overexpressed in neutrophil actin dysfunction syndrome (NAD47/89), a disease characterized by recurrent bacterial infections and immotile neutrophils 220–221. Overexpressed LSP1 alters actin organization, generating F-actin spikes at the cell surface, with impaired chemotaxis, phagocytosis, and cell spreading. However, the genetic basis of the patient described with neutrophil actin dysfunction syndrome has not yet been characterized.

## Candidate genes for actin immunodeficiencies

Seven genes have been identified as causing human actin-related immunodeficiency: *WAS, WIPF1, DOCK8, RAC2, RHOH, CORO1A,* and *ACTB*. Five of these are involved in Rho GTPase-mediated cytoskeletal organization (WASp, WIP, DOCK8, Rac2 and RhoH), Coronin 1A negatively regulates Arp2/3, and β-Actin is the central component of the cytoskeleton. Given that over 100 proteins are directly involved in actin cytoskeletal regulation 5, there is a clear potential for the identification of other actin-related immunodeficiencies. Some examples are shown below.

### RhoG

Mutations in all three key stages of Rho GTPase signaling have been described in immunodeficiency: the effectors (WASp), the GTPases (Rac2 and RhoH), and the upstream modulators (the Rho/Rac GEF DOCK8), suggesting this pathway is particularly important in immune function. The majority of Rho GTPase signaling molecules has a wide tissue distribution and loss of activity has major consequences beyond immune cell function. However, while RhoG is widely expressed and functions in many tissues, it is most highly expressed in lymphocytes 181. *RhoG* KO mice develop normally, have normal numbers of B and T cells, increased IgG1 and IgG2b, and a small increase in B and T-cell proliferation following TCR or B-cell receptor activation 222. RhoG is also important for oxidative burst and both FcγR and complement receptor 3-mediated phagocytosis and has recently been shown to be essential in the internalization of the TCR and bound MHC class II complex 223,224.

### L-plastin

L-plastin is an actin-bundling protein whose expression is restricted to hematopoietic cells 226–227. Knockout of L-plastin in mice revealed an essential role in neutrophil oxidative burst and bacterial killing 228. There are now numerous reports of a variety of murine and cell line immune defects due to L-plastin deficiency. L-plastin plays important roles in both myeloid and lymphoid lineages, in receptor signaling, adhesion, and motility (reviewed in 229), and as such is a strong candidate for human immunodeficiency.

### AIP1

In mice, knockout of the Actin-interacting protein 1 gene (*AIP1*) is embryonic lethal, but hypomorphic mutation results in macrothrombocytopenia and autoinflammatory disease characterized by excessive neutrophil recruitment to inflammation sites 230. AIP1 is ubiquitously expressed and promotes actin filament disassembly via cofilin 231. The precise mechanism for AIP1 actin severing is not known. AIP1 alone has little effect on actin dynamics, but in the presence of cofilin, AIP1 may directly sever actin filaments 232,233, and/or cap the barbed ends of filaments severed by cofilin 233–235. AIP1 activity is further enhanced by interactions with coronin and the immunomodulatory caspase-11 236,237. Depletion of AIP1 in the Jurkat T-cell line impairs migration through generation of multiple membrane protrusions rather than the single lamellipodium required for directed migration 239. *AIP1* KO neutrophils show increased levels of F-actin, but a loss of cortical F-actin, cofilin mislocalization, and reduced migration 230. Partial loss of AIP1 function may result in similar immune dysregulation in humans.

### MKL1

Megakaryoblastic leukemia myocardin-like 1 (MKL1/MAL/MRTF-A) is an actin-binding co-factor that senses G-actin concentration and is a co-activator of the transcription factor serum response factor (SRF) 240. Over 100 SRF target genes have actin cytoskeletal functions, of which 28 are directly dependent on MKL1 activity 241–242. Knockout of *MKL1* in mice results in megakaryocyte maturation defects and thrombocytopenia, and may therefore mimic some aspects of WAS 243,244.

## Summary and conclusions

Immunodeficiencies due to actin cytoskeletal defects are so far rare, but their study has provided valuable insight into the normal function of the cytoskeleton in the immune system. The fact that defects in a number of genes are known to give rise to actin-related immunodeficiencies serves to highlight the importance of the cytoskeleton in normal immune homeostasis and effector function. It is therefore highly likely that further genes will be identified as the cause of actin-related immunodeficiencies.
